# *Nauclea officinalis*: A Chinese medicinal herb with phytochemical, biological, and pharmacological effects

**DOI:** 10.1186/s13020-022-00691-8

**Published:** 2022-12-20

**Authors:** Bin Liu, Qi Geng, Zhiwen Cao, Li Li, Peipei Lu, Lin Lin, Lan Yan, Cheng Lu

**Affiliations:** Institute of Basic Research in Clinical Medicine, China Academy of Chinese Medical Sciences, Dongcheng District, Beijing, 100700 China

**Keywords:** *Nauclea officinalis*, Phytochemistry, Biological activity, Pharmacological effect

## Abstract

*Nauclea officinalis* (*N. officinalis*), a medicinal plant of the genus *Nauclea* in the family Rubiaceae, is used in the treatment of fever, pneumonia, pharyngolaryngitis, and enteritis in China. Extracts of *N. officinalis* include alkaloids, phenolic acids, pentacyclic triterpenoids, and flavonoids, which exert all kinds of pharmacological effects, for instance anti-inflammatory, anti-tumor, antibacterial, and antiviral and therefore show good effectiveness. To gain a comprehensive and deep understanding, the medicinal chemistry and chemical biology of *N. officinalis* are summarized in this review to provide a theoretical basis. The pharmacological effects were reviewed to provide evidence or insights into potential opportunities for further studies and medicinal exploitation of *N. officinalis*.

## Introduction

*Nauclea officinalis* (*N. officinalis*), also known as Dan Mu, is a traditional herbal medicine distributed in Hainan, Yunnan, and Guangxi in China [1]. *N. officinalis* belong to the genus *Nauclea* in the family Rubiaceae [2, 3]. A large majority of these are woody trees and shrubs that are primarily found in tropical regions [4]. In Li folk medicine, *N. officinalis* has been used to treat inflammatory and infectious diseases including diarrhea, pneumonia, and enteritis [5–7].

*N. officinalis* exhibits various potential bioactivities such as anti-inflammatory [8], suppression of tumor cell growth [9], antibacterial [10], antiviral [11], and antimalarial [12]. As a result, *N. officinalis* extracts  were used in traditional herbal medicine for many years to treat acute tonsillitis and upper respiratory tract infection [13]. In addition, various chemical components, including alkaloids, pentacyclic triterpenoids, and phenolic acids, were isolated and identified by different researchers from *N. officinalis* [6, 7, 12]. According to studies, *N. officinalis*' chemical components have several beneficial properties with anti-inflammatory, anti-tumor, antibacterial, and antiviral activities [14–16].

Alkaloids are the main secondary metabolites found in *N. officinalis*, which are heterocyclic molecules containing at least one nitrogen atom in their structure [17]. As the main classification of alkaloids, indole alkaloids, which have antibacterial, antimalarial, antifungal, antiparasitic, anti-inflammatory, antiviral, antineoplastic, anti-acethylcolinesterase, and anti-buthyrylcolinesterase properties [18, 19], are chemical components of *N. officinalis.* For example, strictosamide (STR) and vincosamide are the main active components of *N. officinalis* indole alkaloids [20]. STR is the most abundant constituent of indole alkaloids and has a variety of pharmacological effects, such as anti-inflammatory properties, antipyretic properties, and antiviral properties [21]. Vincosamide is an isomer of STR that exhibits anti-tumor [22], anti-inflammatory [23], and other properties. According to pharmacokinetic studies, these two compounds are readily absorbed into the plasma and exhibit good pharmacological effects.

The compounds of quinoline alkaloids have N-based heterocyclic aromatic and have a broad range of biological activities including anti-tumor, antiparasitic, antibacterial, cardioprotective, antiviral, anti-inflammatory, antioxidant, and antitussive activities [24, 25]. Triterpenoids make up this diverse class of natural plant products, which contain three terpene units and are effective against tumors, inflammation, and viruses [26]. Phenolic acids are phenolic compounds that are resonance stabilized and possess a phenol moiety. As a result, antioxidant properties are achieved through radical scavenging mechanisms that lead to H-atom donation. Furthermore, phenolic acids have health protective effects, for instance anti-microbial, anti-tumor, anti-inflammatory, and anti-mutagenic effects [27]. These findings indicate the potential medicinal value of *N. officinalis* as a Chinese herbal medicine.

Our review summarizes phytochemical, bioactive, and pharmacological effects of *N. officinalis*, providing useful information for its development and utilization.

## Phytochemistry and biological activity of *N. officinalis*

Numerous types of chemical constituents have been identified in *N. officinalis*, including alkaloids, pentacyclic triterpenoids, and phenolic acids, according to phytochemical analysis (Table 1). Alkaloids are the characteristic components of *N. officinalis* and its main active ingredients.

**Table 1:** The phytochemistry and biological activity of *N. officenalis*

No	Identification compound	Source	Category	Molecular formula	Structural formula	biological activity
1	Naucleficine [30]	Stems	Indole alkaloid	C_20_H_14_N_2_O_2_		N/A
2	Nauclefidine [30, 36, 37]	Stems	Indole alkaloid	C_16_H_12_N_2_0_2_		Anti-inflammatory antivirus
3	Nauclefoline [30]	Stems	Indole alkaloid	C_19_H_16_N_2_0_3_		N/A
4	Naucleaoffines A [37]	Stems leaves	Indole alkaloid	C_20_H_24_N_2_O_3_		Anti-inflammatory antivirus
5	Naucleaoffines B [37]	Stems leaves	Indole alkaloid	C_20_H_24_N_2_O_3_		Anti-inflammatory antivirus
6	3,14-Dihydroangustine [37, 47]	Stems Leaves	Indole alkaloid	C_20_H_17_N_3_O		Anti-inflammatory Antivirus
7	3,14,18,19-Tetrahydroangustine [37]	Stems Leaves	Indole alkaloid	C_20_H_17_N_3_O		Anti-inflammatory Antivirus
8	Angustoline [12, 40, 42, 47, 48]	Stems Bark	Indole alkaloid	C_20_H_17_N_3_O_2_		Anti-inflammatory Anti-tumor Anticholinesterase
9	Angustine [37, 40–42, 47, 48]	Bark	Indole alkaloid	C_20_H_15_N_3_O		Anti-inflammatory Antivirus Vasorelaxant Anticholinesterase
10	Naucletine [37, 40, 41]	Bark	Indole alkaloid	C_20_H_15_N_3_O_2_		Anti-inflammatory Antivirus Vasorelaxant Anticholinesterase
11	Naucleofficine H [11]	Stems	Indole alkaloid	C_20_H_22_N_2_O_2_		Anti-inflammatory Antivirus
12	Strictosamide [11, 14, 28, 29, 31, 35, 36, 47–52]	Stems	Indole alkaloid	C_26_H_30_N_2_O_8_		Anti-inflammatory Antivirus Anti-microbial Antimalarial Analgesic Anticholinesterase
13	Vincosamide [14, 28, 29, 35, 36, 48, 50]	Stems	Indole alkaloid	C_26_H_30_N_2_O_8_		Anti-tumor Anti-inflammatory Anti-microbial Antimalarial
14	Naucleactonin A [2, 37]	Bark Wood	Indole alkaloid	C_19_H_14_N_2_O_3_		Anti-inflammatory Antivirus
15	Naucleactonin B [2]	Bark Wood	Indole alkaloid	C_20_H_18_N_2_O_4_		N/A
16	(E)-2-(1-*β*-D-glucopyrano-syloxybut-2-en-2-yl)-3-(hydroxymethyl)-6,7-dihydro-indolo[2,3-a] quinolizin-4(12H)-one [12]	Stems Bark	Indole alkaloid	C_26_H_29_N_2_O_8_		Antimalarial
17	(E)-1-propenyl-12-*β*-D-glucopyranosyloxy-2,7,8-trihydro-indolo[2,3-a]pyran[3,4-g]quinolizin-4,5(13H)-dione [12]	Stems Bark	Indole alkaloid	C_26_H_26_N_2_O_9_		Antimalarial
18	(E)-2-(1-hydroxybut-2-en-2-yl)-11-*β*-D-glucopyranosyloxy-6,7-di-hydro-indolo[2,3-a]quinolizin-4(12H)-one [12]	Stems Bark	Indole alkaloid	C_25_H_28_N_2_O_8_		Antimalarial
19	Naucleofficine III [7, 53]	Stems Bark	Indole alkaloid	C_20_H_22_N_2_O_3_		Anti-tumor
20	1-(1-hydroxyethyl)-10-hydroxy-7,8-dihydro-indolo[2,3-a]pirydine[3,4-g]quinolizin-5(13H)-one(10-hydroxyangustoline [12]	Stems Bark	Indole alkaloid	C_20_H_17_N_3_O_3_		Antimalarial
21	Naucleidinal [7, 12, 36]	Stems Bark	Indole alkaloid	C_20_H_21_N_2_O_3_		Anti-tumor
22	Naucline [7, 41, 54]	Bark Stems Leaves	Indole alkaloid	C_20_H_18_N_2_O_2_		Vasorelaxant Anticholinesterase
23	Angustidine [40, 41]	Bark	Indole alkaloid	C_19_H_15_N_3_O		Anticholinesterase
24	Nauclefine [40–42, 47]	Bark	Indole alkaloid	C_18_H_13_N_3_O		Anti-inflammatory Vasorelaxant Anticholinesterase
25	1,2,3,4-tetrahydro-1-oxo-*β*-carboline [42]	Twigs	Indole alkaloid	C_11_H_12_N_2_		Anti-inflammatory
26	3*β*,19*α*,23,24-tetrahydroxyurs-12-en-28-oic acid [46]	Stems	Pentacyclic triterpenoid	C_30_H_48_O_6_		Anti-inflammatory
27	2*β*,3*β*,19*α*,24-tetrahydroxyurs-12-en-28-oic acid [46]	Stems	Pentacyclic triterpenoid	C_30_H_48_O_6_		Anti-inflammatory
28	Naucleamide G [7, 28, 38]	Stems Leaves	Indole alkaloid	C_25_H_32_N_2_O_8_		Anti-inflammatory
29	Nauclealomide B [38]	Leaves	Indole alkaloid	C_26_H_30_N_2_O_10_		N/A
30	Nauclealomide C [38]	Leaves	Indole alkaloid	C_26_H_30_N_2_O_10_		N/A
31	3*α*,5*α*-tetrahydrodeoxycordifoline lactam [29]	Stems	Indole alkaloid	C_27_H_30_N_2_O_10_		Anti-inflammatory
32	Naucleamide A-10-O-*β*-D-glucopyranoside [28, 29, 36, 47, 55]	Stems	Indole alkaloid	C_26_H_34_N_2_O_9_		Anti-inflammatory
33	Paratunamide C [38]	Leaves	Indole alkaloid	C_26_H_30_N_2_O_11_		N/A
34	Paratunamide D [38]	Leaves	Indole alkaloid	C_26_H_30_N_2_O_11_		N/A
35	Paratunamide A [38]	Leaves	Indole alkaloid	C_26_H_30_N_2_O_11_		N/A
36	17-O-methyl-19-(Z)-naucline [31, 39]	Stems Leaves	Indole alkaloid	C_21_H_20_N_2_O_3_		Anti-inflammatory
37	17-oxo-19-(Z)-naucline [42]	Bark Twigs	Indole alkaloid	C_20_H_16_N_2_O_3_		Anti-inflammatory
38	Naucleoffieine H [31]	Stems Bark	Indole alkaloid	C_20_H_22_N_2_O_3_		Anti-inflammatory
39	Pumiloside [11, 28, 29, 36, 47, 48, 50, 56]	Stems	Quinoline alkaloid	C_26_H_28_N_2_O_9_		Anti-inflammatory Anti-microbial Antivirus
40	3-epi-pumiloside [14, 29, 36, 50]	Stems	Quinoline alkaloid	C_26_H_28_N_2_O_9_		Anti-inflammatory Anti-microbial
41	Pyrocincholic acid 3*β*-O-*α*-L-rhamnopyranoside [9]	Stems	Pentacyclic triterpenoid	C_35_H_56_O_7_		Anti-tumor
42	Pyrocincholic acid 3*β*-O-*α*-L-rhamnopyranosy1-28-O-*β*-D-glucopyranosyl-(1–6)-*β*-D-glucopyranosyl ester [9]	Stems	Pentacyclic triterpenoid	C_47_H_76_O_17_		Anti-tumor
43	Pyrocincholic acid 3*β*-O-*α*-L-rhamnopyranosy1-28-O-*β*-D-glucopyranosyl ester [9]	Stems	Pentacyclic triterpenoid	C_41_H_66_O_12_		Anti-tumor
44	Protocatechuic acid [35, 36]	Stems	Phenolic acid	C_7_H_5_O_4_		Antioxidation Anti-inflammatory Anti-microbial
45	Chlorogenic acid [35, 36]	Stems	Phenolic acid	C_16_H_17_O_9_		Antioxidation Anti-inflammatory Anti-microbial

### Alkaloids

Alkaloids are the most widely reported class of compounds found in *N. officinalis*. The alkaloids in *N. officinalis* are mainly indole alkaloids; however, a few quinoline alkaloids are also present. Studies have shown that certain indole alkaloids exhibit anti-inflammatory, antibacterial, and antiplasmodial activity.

#### Indole alkaloids

The indole alkaloids in *N. officinalis* have the following characteristics: the structural skeleton generally consists of five six-membered rings; indoles (A/B ring) and tetrahydropyridines (C ring) form the terahydro-*β*-carboline parent ring, the D ring is a saturated or unsaturated lactam ring, the E ring can be an azapyridine or oxatetrahydrofuran ring, and some compounds have an open or non-existent E ring [12].

Chemical constituents extracted from the stems of *N. officinalis* are STR [14, 28, 29], vincosamide [14, 28, 29], naucleficine [30], nauclefidine [30], nauclefoline [30], naucleofficine H [11, 31], naucleamide A-10-O-*β*-D-glucopyranoside [28, 29], and 3*α*,5*α*-tetrahydrodeoxycordifoline lactam [29]. STR is a representative component of indole alkaloids, which are present in all plants of the genus *Nauclea*, at high concentrations, and is also the main active ingredient extracted from *N. officinalis*. Therefore, STR is mostly used in studies as a standardized assay for the quality of extracts from plants of the genus *Nauclea* with various biological activities, for instance anti-inflammatory, antibacterial, antiviral, analgesic, anti-tumor, and antimalarial [32–34]. Alternatively, vincosamide, an isomer of STR, assists in reducing inflammation, bacteria proliferation, and malaria transmission [35, 36]. Naucleofficine H, a colorless crystal, can stimulate the proliferation of human umbilical vein endothelial cells (HUVECs) [11] and has anti-inflammatory activity [31]. nauclefidine [30], 3*α*,5*α*-tetrahydrodeoxycordifoline lactam [29], and naucleamide A-10-O-*β*-D-glucopyranoside [29] also have anti-inflammatory activities.

As natural extracts of *N. officinalis* stems and leaves, naucleaoffine A and naucleaoffine B, as well as 3,14-Dihydroangustine and 3,14,18,19-Tetrahydroangustine [37], are yellowish amorphous powders inhibiting inflammation and HIV-1 replication. Naucleamide G is an orange amorphous powder [28, 38], and 17-O-methyl-19-(Z)-naucline is a yellowish amorphous powder with anti-inflammatory activity [31, 39]. Nauclealomide B, nauclealomide C, paratunamide A, paratunamide C, and paratunamide D is extracted from the leaves of *N. officinalis* [38].

Angustine, naucletine, harmane, angustidine and nauclefine, and indole nlkaloids from the bark of *N. officinalis* have anticholinesterase bioactivities [40]. Angustine, nucletine, and nauclefine also have anti-inflammatory, antivirus, and vasorelaxant activities [41]. Angustoline, naucleofficine III, naucleidinal, (E)-2-(1-*β*-D-glucopyrano-syloxybut-2-en-2-yl)-3-(hydroxymethyl)-6,7-dihydro-indolo[2,3-a] quinolizin-4(12H)-one, (E)-1-propenyl-12-*β*-D-glucopyranosyloxy-2,7,8-trihydro-indolo[2,3-a]pyran[3,4-g]quinolizin-4,5(13H)-dione, (E)-2-(1-hydroxybut-2-en-2-yl)-11-*β*-D-glucopyranosyloxy-6,7-di-hydro-indolo[2,3-a]quinolizin-4(12H)-one, and 1-(1-hydroxyethyl)-10-hydroxy-7,8-dihydro-indolo[2,3-a]pirydine[3,4-g]quinolizin-5(13H)-one(10-hydroxyangustoline), extracts of *N. officinalis* stems and bark are weak to moderately effective against *Plasmodium falciparum* [12]. Angustoline also exhibits significant cytotoxic [12] and anti-inflammatory activities [42].

Phytochemicals such as nucleactonin A and B are synthesized from *N. officinalis* bark and wood [2]. Only naucleactonin A has been shown to exhibit anti-inflammatory activity [37]. 1,2,3,4-tetrahydro-1-oxo-*β*-carboline and 17-oxo-19-(Z)-naucline are isolated from twigs of *N. officinalis*. They show weak anti-tumor activity and significant anti-inflammatory activity [42]. Naucline is isolated as a brownish amorphous solid from the bark, stems, and leaves of *N. officinalis*, and exhibit potent vasorelaxant activity [41].

#### Quinoline alkaloids

Another class of N-based heterocyclic compounds is quinazoline alkaloids. There are about 150 quinazoline alkaloids found in plants, animals, and microorganisms that occur naturally. Several them are genetically descended from anthranilic acid [24]. The quinolone alkaloids pumiloside and 3-epi-pumiloside, extracted from the stems, are among the main active components of *N. officinalis* [43], are present in high amounts, and exhibit various effects, such as anti-inflammatory, antibacterial, and anti-tumor activities [7, 44, 45].

### Pentacyclic triterpenoids

Pentacyclic triterpenes are also important components of *N. officinalis*. Structurally, five or six members are present in ring E of pentacyclic triterpenoids, while A, B, C, and D are six-membered rings. There are six subgroups of carbon skeletons: hopane, ursane, friedelane, gammacerane, lupane, and oleanane [26]. The stems of *N. officinalis* were extracted, 3*β*,19*α*,23,24-tetrahydroxyurs-12-en-28-oic acid and 2*β*,3*β*,19*α*,24-tetrahydroxyurs-12-en-28-oic acid are colorless orthorhombic crystals with significant and weak anti-inflammatory activities [46]. Pyrocincholic acid 3*β*-O-*α*-L-rhamnopyranoside, pyrocincholic acid 3*β*-O-*α*-L-rhamnopyranosy1-28-O-*β*-D-glucopyranosyl-(1–6)-*β*-D-glucopyranosyl ester, and pyrocincholic acid 3*β*-O-*α*-L-rhamnopyranosy1-28-O-*β*-D-glucopyranosyl ester are white amorphous powders and show promising anti-tumor activity [9].

### Phenolic acids

Two main subgroups of phenolic acids with one carboxylic acid group can be distinguished: hydroxybenzoic and hydroxycinnamic acids [27]. Extracted from the stems of *N. officinalis*, protocatechuic acid [35, 36], chlorogenic acid [35, 36], 3,4-dimethoxyphenol-*β*-D-apiofuranosyl (1–6)*β*-D-glucopyranoside [14], and kelampayoside A [14, 36] exhibit excellent antioxidant, anti-inflammatory, and anti-microbial activities, and these factors are likely to contribute to N. officinalis' clinical effectiveness [14, 35, 36].

## Pharmacological effect of *N. officinalis*

### Anti-inflammatory effect

#### In vitro experiment

Experiments on N. officinalis leaf extract have shown anti-inflammatory activity in vitro, which may inhibit the cellular inflammatory response by inhibiting prostaglandin E2 production and release and inhibiting cyclic adenosine monophosphate-specific phosphodiesterase 4 activity [57, 58]. Inflammatory protein (nitric oxide [NO]) and TNF-*α* overproduction have been down-regulated by Naucleoffieine H through induction of lipopolysaccharide (LPS)-induced RAW 264.7 by blocking inducible nitric oxide synthase (iNOS) [31]. Naucleaoffines A and B, naucleactonin A, nauclefidine, 3,14-dihydroangustine, 3,14,18,19-tetrahydroangustine, angustine, and naucletine exhibited significant inhibitory activities against inflammation in RAW 264.7 in vitro [37]. 17-O-methyl-19-(Z)-naucline significantly inhibited NO production in RAW 264.7. No cytotoxicity was observed in 17-O-methyl-19-(Z)-naucline-treated cells [39]. 17-oxo-19-(Z)-naucline, angustoline, angustine, nauclefine and 1,2,3,4-tetrahydro-1-oxob-carboline showed that it inhibited LPS-stimulated NO production in RAW264.7 in vitro [42]. Tao et al. [46] examined the inhibitory effects of 3*β*, 19*α*, 23, 24-tetrahydroxyurs-12-en-28-oic acid and 2*β*, 3*β*, 19*α*, 24-tetrahydroxyurs-12-en-28-oic acid on the production of NO induced by LPS in RAW 264.7 to test their anti-inflammatory effects. They exhibited inhibitory activity on the production of NO with the IC_50_ values of 4.8 and 26.2 mM, respectively. Angustuline, an indole alkaloid found in *N. officinalis*, has anti-inflammatory effects on LPS-induced RAW 264.7 by downregulating the expression of iNOS inflammatory protein, thus inhibiting the production of NO by macrophages [59].

Moreover, STR, pumiloside, 3-epi-pumiloside, vincosamide, 3*α*,5*α*-tetrahydrodeoxycordifoline lactam and naucleamide A-10-O-*β*-D-glucopyranoside exhibited significant inhibitory activity on inflammation [29, 50]. STR, the major compound in *N. officinalis* extract, significantly reduced pro-inflammatory mediator production including NO and cytokines. Additionally, it suppressed iNOS and phosphorylation of I*κ*B*α* and NF-*κ*B p65 in the NF-*κ*B and mitogen-activated protein kinase (MAPK) signaling pathways [29, 50].

#### In vivo experiment

*N. officinalis* is a strong modulator of inflammatory immune responses and suppresses infection and development during the initial stages of infection and inflammation. Asthma mice can be regulated by *N. officinalis* affecting the secretion of cytokines, such as interferon gamma (IFN-*γ*), interleukin (IL) -10, IL-5, IL-4, and IL-2, and the infiltration of inflammatory cells in the airway [60]. *N. officinalis* leaves purified by alcohol extraction-macroporous resin inhibited systemic and local inflammatory responses in rats with acute pharyngitis [61]. Acute inflammation occurs at the beginning, *N. officinalis* extract tablets inhibited exudation, swelling, and late granulation tissue formation in animals with an inflammatory response model. In addition, *N. officinalis* injection inhibited inflammatory cell infiltration in asthmatic mice, thereby improving bronchial asthma [62]. STR is the main representative constituent of *N. officinalis* and contributes significantly to ameliorate inflammation by downregulating the expression of pro-inflammatory cytokines, including tumor necrosis factor (TNF-*α*), IL-1*β*, and IL-6, and inhibiting the NF-*κ*B signaling pathway [63]. A significant reduction in the edema caused by terephthalic acid (TPA) was observed at 20 and 40 mg/kg of STR, and a significant reduction in mouse peritoneal vascular permeability in response to acetic acid. Furthermore, STR significantly reduced leukocyte numbers within the mouse peritoneum induced by sodium carboxymethyl cellulose (CMC-Na). In 20 mg and 40 mg/kg doses of STR, pain latency was markedly prolonged, and the number of writhes was decreased when 40 mg/kg was used [14].

### Anticancer effect

Pyrocincholic acid 3*β*-O-*α*-L-rhamnopyranoside, pyrocincholic acid 3*β*-O-*α*-L-rhamnopyranosy1-28-O-*β*-D-glucopyranosyl-(1–6)-*β*-D-glucopyranosyl ester, and pyrocincholic acid 3*β*-O-*α*-L-rhamnopyranosy1-28-O-*β*-D-glucopyranosyl ester was presented a promising cytotoxic effect against A549 cells [9]. The cytotoxic effects of nautiloids and angustoline have been shown against several human cancer lines, including the breast prostate PC3, leukemic K562, leukemic HL-60, lung A549, as well as the gastro SGC 7901 [12]. In a study with cells of human colon cancer, human gastric cancer, and human hepatoma, nuclear antibiotic III was found to be active against them [53]. In the presence of five human cancer cell lines: HepG2, A549, KB, MCF-7, and K562, STR exhibited moderate antiproliferative properties [15]. In Nauclea roots, two new indole alkaloids, naucleaorals A and B, were isolated. Testing their cytotoxicity for human cervical cancer (HeLa) and human oral epidermoid carcinoma (KB) revealed that with an IC_50_ value of 4.0 g/L, Naucleaorals A exhibited significant cytotoxicity to HeLa cells,, whereas a modest cytotoxicity was found for naucleaorals B against both cell lines with IC_50_ values of 7.8 and 9.5 µg/L, respectively [64].

### Anti-microbial effect

He et al. [10] observed the sensitivity of *Escherichia coli* to *N. officinalis* decoction through an in vitro antibacterial test. The results showed that *N. officinalis* has a bacteriostatic effect on clinically isolated urinary *Escherichia coli*. Xu et al. [65] found that the minimum inhibitory concentration (MIC) and minimum bactericidal concentration (MBC) of *N. officinalis* extract against *Staphylococcus aureus* ranged 1.56–3.13% and 1.56–25%, respectively. *Staphylococcus aureus* has good antibacterial and bactericidal activities against both clinically resistant and sensitive strains, and its MIC to bacteria is close to MBC, suggesting that its antibacterial activity may be related to its direct bactericidal activity. Su et al. [66] detected the antibacterial activity of volatile components using the method of paper-disk diffusion. Results revealed that the volatile components of *N. officinalis* leaves and stems had strong antibacterial effects in Methicillin-resistant *Staphylococcus aureus*, *Escherichia coli*, *Bacillus subtilis*, and *Proteus*. It has also been reported that *N. officinalis* has significant bactericidal and antibacterial effects on bacteria such as *Streptococcus pneumoniae*, *Haemophilus influenzae*, *Streptococcus haemolyticus*, and *Shigella dysenteriae*. Jiang et al. [67] showed that the combined antibacterial effect of *N. officinalis* extract and penicillin showed synergistic action and had an efficient inhibitory effect on *S. aureus*.

### Antiviral effect

Children were treated with *N. officinalis* and ribavirin injections for acute upper respiratory tract infections. The results showed that *N. officinalis* injection inhibited bacterial and viral protein synthesis, and metabolism of folic acid is blocked. The clinical effect of *N. officinalis* is more significant than that of ribavirin injection and has the advantages of broad-spectrum antibacterial, anti-virus, and less susceptibility to drug resistance [61]. N. officinalis stems and leaves are rich in monoterpene indole alkaloids, which exhibit significant anti-HIV-1 activities, with IC_50_s ranging from 0.06–2.08 µM. *N. officinalis* may be a valuable source of indole alkaloids with significant anti-HIV-1 activities that could be developed into new anti-HIV agents. [37]. The anti-HIV-1 activities of Naucleaoffines A and B, naucleactonin A, nauclefidine, 3,14-dihydroangustine, 3,14,18,19-tetrahydroangustine, angustine, and naucletine were, with EC_50_ ranged between 0.06 and 2.08 μM [37]. Biological studies confirmed this activity, by measuring the IC_50_ values of 25.68 g/mL for influenza A virus and 12.50 g/mL for respiratory syncytial virus [15].

### Other pharmacological effects

The ingredients of *N. officinalis* also have antimalarial, vasorelaxant, and anticholinesterase effects, promoting the proliferation of HUVEC and antihypertensive pharmacological effects. In vitro, (E)-2-(1-b-D-glucopyranosyloxybut-2-en-2-yl)-3-(hydroxymethyl)-6, 7-dihydro-indolo [2, 3-a] quinolizin-4 (12H)-one exhibited potential antimalarial activity against *P. falciparum*, [12]. STR displayed moderate antiplasmodial activity against *P. falciparum* [68].

The vasorelaxant effects of naucline, angustine, nauclefine, and naucletine were demonstrated in rat aorta experiments, it may be facilitated by an increase in NO release by endothelial cells [41]. In addition, there was a relaxation induced by naucine independent of endothelial relaxation [54].

In the anticholinesterase activity test, angustidine, nauclefine, angustine, angustoline, and andharmane showed higher butyrylcholinesterase inhibitory potency. Angustidine is known to be one of the greatest inhibitors of acetylcholinesterase and butyrylcholinesterase, which forms hydrogen bonds with Ser198 and His438 [40].

Inflammation can be repaired by proliferating and migrating cells. A previous study found that Naucleofficine H, STR, and pumiloside (0–200 μg/mL) promoted HUVEC proliferation by upregulating vascular endothelial growth factor (VEGF) and phosphorylation-extracellular regulated protein kinases (ERK) in HUVEC [11].

In addition, different concentrations of STR decreased Mg^2+^-ATPase activity in the brain and kidneys in vivo and in vitro. STR also increased Na^+^/K^+^-ATPase activity in the brain in vivo. These results may account for the effect of *N. officinalis* infusions on hypertension [69]. Although there are many studies to explore the pharmacological effects of *N. officinalis* (Table 2), there is still a lack of in-depth research on its mechanism of action, which also indicates that *N. officinalis* and its active ingredients have great research and development potential.

**Table 2:** Pharmacological effect of *Nauclea officinalis* and its different extracts in vivo and in vitro

NO	Compound Name	Cells/ Virus/Bacterials	Animal model	Dose/concentration	Results
1	Naucleoffieine H [31]	RAW264.7	/	3, 10, 30 μM	Reduced productions of NO, iNOS and TNF-*α*
2	Monoterpene indole alkaloids [37]	RAW264.7, HIV-1	/	0.0625, 0.32, 1.6, 8, 40 μM	Reduced productions of NO, inhibited the activity of HIV-1
3	Nauclea officinalis [50]	RAW264.7	/	10, 20, 50, 100 µg/mL	Reduced mRNA expressions and productions of NO, TNF-*α*, IL-6 and IL-1*β*; Suppressed p-I*κ*B*α* and p-p65
4	Strictosamide [29]	RAW264.7	/	0, 25, 50, 100, 200 µM	Suppressed productions of NO, iNOS, TNF-*α* and IL-1*β*; Reduced expressions of p-p65, p-I*κ*Bα, p-IKK*α*, p-p38, p-ERK and p-JNK
5	Strictosamide [14]	/	ICR mice	20, 40 mg/kg	Reduced ear edema in mice induced by TPA; Reduced peritoneal vascular permeability in mice stimulated by acetic acid; Reduced leukocyte count in the peritoneal cavity of mice after CMC-Na treatment; Prolonged the pain latency and decreased the writhing counts
6	Extracts of Nauclea officinalis Pierre ex Pitard [60]	/	Balb/c mice, ovalbumin induced asthma model	1, 2, 4 mg/kg	Reduced the number of inflammatory cells in mouse BALF; Affected the secretion of cytokines in mouse BALF
7	Extracts of Nauclea officinalis Pierre ex Pitard leaf [57]	/	ICR mice	0.390, 0.195, 0.098 g/kg/days	Prolonged pain latency; Reduced the number of twisting reactions and antagonized the increased intra-abdominal capillary permeability in mice caused by acetic acid; Reduced xylene-induced swelling of the pinna in mice
8	Strictosamide [63]	IEC6/HT-29	/	100, 200 μM	Reduced disease activity index and reduced H&E damage in DSS model mice; Reduced the expression of p-I*κ*B*α*, p-p65; Downregulated expressions of TNF-*α*, IL-1*β*, IL-6, MPO and iNOS
		/	Male Balb/c mice, dextran sulfate sodium (DSS) induced ulcerative colitis model	20, 40 mg/kg	
9	Extracts of Nauclea officinalis Pierre ex Pitard [67]	Staphylococcus aureus	/	0.3125, 0.625, 1.25, 2.5, 5, 10, 20, 40, 80, 160, 320 mg/kg	Had an efficient inhibitory effect on Staphylococcus aureus

## Conclusions

As one of the rare wild plant species under key protection in China, *N. officinalis* is also an important medicinal plant. The dry trunk, branches, bark, leaves, and roots of *N. officinalis* can be medicated, and has the effect of clearing heat and detoxification, reducing swelling and relieving pain. The chemical composition of *N. officinalis* mainly includes alkaloids, pentacyclic triterpenes and their saponin compounds, phenolic acids, flavonoids, amino acids, and various trace elements. In recent years, from the consideration of expanding the source of drugs, people have paid more and more attention to composition and biological activity of *N. officinalis*. The content of total flavonoids, total flavone glycosides and alkaloids in *N. officinalis* is high, especially the content of alkaloids is as high as 3.46%, which has high medicinal value and good development potential [70].

**Fig. 1:**
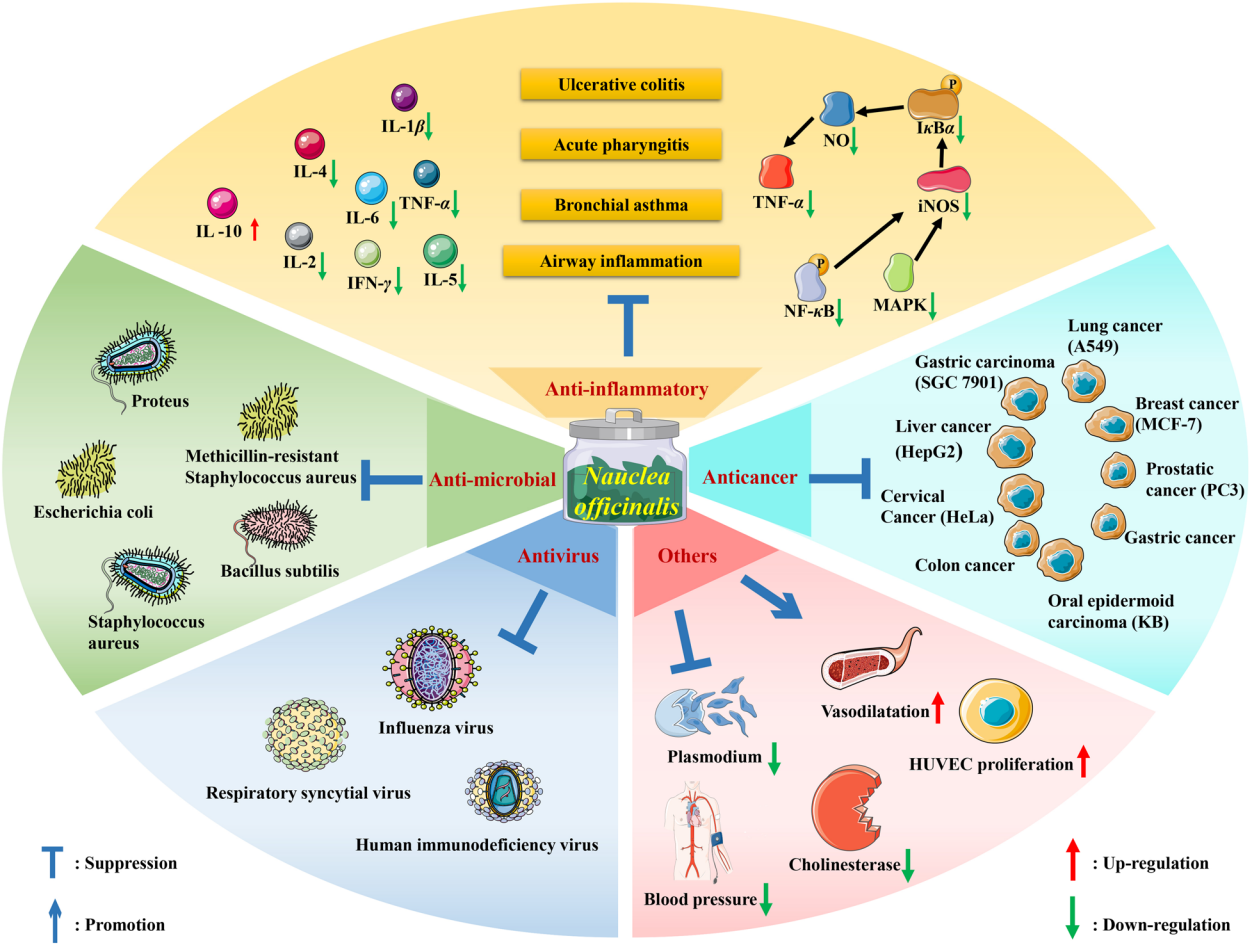
Schematic diagram of pharmacological effect of *N. officinalis*

A variety of pharmacological effects are associated with *N. officinalis*, including anti-inflammatory, anticancer, anti-microbial, and antivirus (Fig. 1). In clinic, *N. officinalis* extract syrup was used to treat acute tonsillitis, pediatric viral influenza, and lower respiratory tract infection with good results [71–73], but more rigorous researches need to be conducted. In some basic research, although a series of anti-inflammatory, anti-tumor, and anti-microbial studies have been conducted for *N. officinalis* and its extracts, these studies only briefly showed their pharmacological effects. In addition, several studies have used extracts of *N. officinalis*, but the specific active ingredient is unclear, which is not conducive to the study of the mechanism of *N. officinalis*. Therefore, exploring the in-depth mechanisms of *N. officinalis*, its extracts, and active ingredients is urgent and may help identify new drugs and targets of drug action. In recent years, with the development and utilization of *N. officinalis* herbs, resources have become increasingly important and the full and rational exploitation of *N. officinalis* resources has become the focus of future research. Meanwhile, developing new preparations or discovering more medicinal value is also an important issue for the development of *N. officinalis*.

## Data Availability

The situation does not apply.

## Abbreviations

*N. officinalis*: *Nauclea officinalis*
STR: Strictosamide
IFN-*γ*: Interferon gamma
IL: Interleukin
TPA: Terephthalic acid
CMC-Na: Sodium carboxymethyl cellulose
NO: Nitric oxide
TNF: Tumor necrosis factor
iNOS: Inducible nitric oxide synthase
LPS: Lipopolysaccharide
MAPK: Mitogen-activated protein kinase
MIC: Minimum inhibitory concentration
MBC: Minimum bactericidal concentration
HUVEC: Human umbilical vein endothelial cells
VEGF: Vascular endothelial growth factor
ERK: Extracellular regulated protein kinases
